# Evaluation of the Early to Mid-term Efficacy and Safety of Deep Sclerectomy without an Intrascleral Spacer for Open-angle Glaucoma in an Australian Population

**DOI:** 10.5005/jp-journals-10028-1254

**Published:** 2018

**Authors:** Michelle M Hui, Colin I Clement

**Affiliations:** 1 Royal Prince Alfred Hospital, Sydney, New South Wales, Australia; 2 Univeristy of Sydney, Sydney, New South Wales, Australia; 3 Eye Associates, Sydney, New South Wales, Australia; 4 Glaucoma Unit, Sydney Eye Hospital, Sydney, New South Wales, Australia; 5 Department of Ophthalmology, University of Sydney, New South Wales, Australia

**Keywords:** Australia, Deep sclerectomy, Glaucoma, Non-penetrating glaucoma surgery, Open-angle glaucoma

## Abstract

**Purpose:**

This study aims to evaluate the early to the midterm efficacy of deep sclerectomy (DS) without an intra-scleral spacer for open-angle glaucoma (OAG) patients.

**Materials and methods:**

Retrospective study of 99 eyes (88 patients) with open-angle glaucoma who underwent DS were recruited in a consecutive order following informed consent. Intraocular pressure (IOP) was collected up to 60 months post operation (mean 19.87 ± 15.13 months). Criteria of success were defined as the qualified success (QS) or complete success (CS) with IOP level less than 21, 18 and 15 mm Hg and a reduction of more than 20% IOP from baseline. QS includes additional medication post-DS, while CS requires no other medications or surgery post-DS. Further analysis includes comparing the criteria of success based on several factors. The data were analyzed using statistical package for social sciences (SPSS version 21) statistical software.

**Results:**

The QS at 60 months for IOP less than 21, 18 and 15 mm Hg is 71.3% (45.12 ± 2.46), 63.9% (40.41 ± 2.75) and 48.7% (35.62 ± 2.85), respectively. The CS at 60 months for IOP less than 21, 18 and 15 mm Hg are 69.3% (47.51 ± 2.77), 57.9% (40.41 ± 2.75) and 45.2% (35.62 ± 2.85), respectively. There was no significant difference between QS and DS post-DS based on the level of experience of the surgeons; intraoperation complication; age and gender. There was a significant reduction in IOP post operation (*p* < 0.001).

**Conclusion:**

DS is observed to be an effective surgical method with a favorable safety profile to manage patients with open-angle glaucoma. It has a better safety profile compared to trabeculectomy (TE) and can be performed by surgeons of different experience safely and successfully.

**Clinical significance:**

To our knowledge, this is the first report of DS in an Australian population with up to 60 months of follow-up. It is an effective procedure for IOP control in patients with OAG and has fewer complications compared to TE. DS is less popular than TE primarily due to a perceived steep learning curve, but most of the literature on DS describe single surgeon results. Our study compared the outcome of five surgeons with a variety of experience and found no significant differences in the rate of success for all levels of IOP.

**How to cite this article:**

Hui MM, Clement CI. Evaluation of the Early to Mid-term Efficacy and Safety of Deep Sclerectomy without an Intrascleral Spacer for Open-angle Glaucoma in an Australian Population. J Curr Glaucoma Pract 2018;12(3):107-112.

## INTRODUCTION

Glaucoma is an optic neuropathy and the second leading cause of blindness worldwide. An estimate of 37 million people worldwide are blind due to glaucoma based on the population-based World Health Organization (WHO) survey in 2002.^1^ The aim of glaucoma management is reducing IOP; methods include surgery, laser, and medication. Surgery is indicated when there is insufficient control from non-surgical methods, or non-surgical methods are poorly tolerated or contraindicated. The TE was first described in 1968 by Cairns et al. for the reduction of IOP by increasing aqueous outflow into the subconjunctival space via a sclerocorneal fistula covered by a partial thickness scleral flap.^2^ Despite improvements in technique (Moorfields Safe Surgery System),^3^ TE is associated with significant and potentially blinding complications which include hypotony and endophthalmitis.^4–7^

With the aim of improving the safety of glaucoma surgery, nonpenetrating surgery (NPS) was developed. DS is one type of NPS technique, first described by Fedorov in 1982,^8^ that shares many similarities to TE, but importantly does not involve a full-thickness penetration into the eye. Instead, Schlemm's canal is de-roofed, but the inner wall of Schlemm's canal is left intact, so resistance to aqueous outflow is preserved. DS has been associated with lower rates of certain serious complications compared to TE including hypotony and endophthalmitis.^4–7^

Despite TE having a higher incident of postoperative complications compared to DS, it remains the gold standard surgical glaucoma treatment with a proven track record and well-documented efficacy. DS is considered more challenging in technique compared to TE and requires a longer learning curve.^6,7,9^ Surgical expertise is one factor that may influence the procedure's outcome, although this has not been studied in depth. Many of the studies on DS are performed by single surgeons and have not described the expertise of the surgeon.^6^ Direct comparison of surgical outcomes performed by fellows and consultants for DS are uncommon.^9^

In this study, we aim to assess the early to the midterm efficacy and safety of DS for OAG in an Australian population. Furthermore, we aim to evaluate the outcome of DS based on expertise (four fellows *vs.* a consultant). To our knowledge, this is the first report of DS outcomes in an Australian population.

## MATERIALS AND METHODS

### Study Population

A retrospective study was conducted of DS outcomes in patients that had surgery in consecutive order from March 2008 to August 2016 from four clinics in Sydney. The study was approved by the Royal Australian and New Zealand College of Ophthalmology Human Research Ethics Committee.

In the study, the demographics and clinical characteristics included were—age, gender, best-corrected visual acuity (BCVA); IOP; glaucoma medications, central corneal thickness, Humphrey visual field analyzer mean deviation, cup to disc ratio, type of glaucoma [primary open angle glaucoma (POAG), secondary open-angle glaucoma (SOAG)], Intra- and post-operative complications. Further measurements (IOP and BCVA) were taken at different intervals post-DS (day 1; week 1; week 4; week 6; week 12; 6 months; 12 months; 18 months; 24 months; 30 months; 36 months; 42 months; 48 months; 54 months and 60 months). A consultant or one of four glaucoma fellows performed all DS procedures in this study.

### Surgical Method

A standard DS technique was used. However, mid-way through the study period, a new method for applying mitomycin C (MMC) was adopted. Earlier cases had MMC 0.02% applied on a subconjunctival sponge with 30-60 second exposure followed by balanced saline washout, whereas later cases had MMC injected into the Tenon's capsule (0.02%, 0.1 mL) before conjunctival peritomy. In all other respects, the surgical technique was the same for all cases. In brief, the superior conjunctival peritomy was large and included a single radial limbal conjunctival relaxing incision. A 5 × 5 mm superficial one-third thickness scleral flap was dissected followed by a 3 × 3 mm deeper flap that extended forward until Schlemm's canal was de-roofed. The deep flap was amputated, and the exposed juxtacanalicular layer of the inner wall of Schlemm's canal was stripped using capsulorhexis forceps. The superficial scleral flap was closed with two 10-0 nylon sutures, Healon GV was injected under the scleral flap, and the conjunctiva was closed using 8–0 Vicryl. Inferior subconjunctival dexamethasone and cefazolin were given at the end of the case.

### Statistical Analysis

The primary outcome of the study was the reduction in IOP following surgery. Surgery was considered successful if (i) IOP was reduced more than 20% from the pre-surgery baseline level, and (ii) IOP was less than a specified target IOP. For analysis, 3 IOP targets were chosen for comparison: < 21, < 18 and < 15 mm Hg. Success was further categorized as either qualified (IOP medication required) or complete (no IOP-lowering medication required). If an eye did not meet the above criteria at two consecutive follow-up visits, the surgery was considered a failure.

Kaplan-Meier survival analysis was performed comparing the baseline IOP to the post-DS IOP based on the success definition as mentioned above. BCVA, number of medications, and IOP reported after surgery were assessed. A paired sample t-test compared the BCVA, some medication, and IOP preoperation to post-DS. Furthermore, Mantel-Cox test was performed to measure the impact of many variables on the IOP reduction following surgery. These variables were the surgeon's expertise, patient's gender, type of glaucoma and occurrence of intraoperative complications. All analyses were conducted with the SPSS version 21 with statistical significance set at *p* < 0.05.

## RESULTS

A total of 99 eyes (88 patients) with OAG were enrolled in the study. Nine patients were lost to follow-up and were not included in the final analysis. The mean follow-up was 19.87 ± 15.13 months. The mean age was 71.78 years (SD 15.67), and 48.3% (n = 42) were female. Intraoperatively, three eyes (2.94%) sustained perforation and were converted to TE. These cases were excluded from the analysis. The 41 (49.5%) eyes goniopuncture post-DS with a mean time of 9.1 ± 6 months between DS and goniopuncture. Table 1 describes the baseline characteristics of the patients.

**Table 1:** Patient baseline characteristics (n = 99 eyes)

*Age*	*71.78* ± *15.67*
Gender (M:F)	51:48
Mean LogMAR visual acuity	0.57 ± 0.42
Mean pre-operation intraocular pressure (mm Hg)	27.66 ± 9.47
Mean medications for glaucoma	3.41 ± 0.99
Mean central corneal thickness	534,28 ± 54.23
Mean Humphrey visual field analyser (mean deviation)	−13.37 (SD 9.27)
Mean cup to disc ratio	0.79 ± 0.23
*Glaucoma type*	
POAG	59
PXFG	17
Uveitis	14
Pigment dispersion	2
Steroid induced	3
Anti-VEGF	1
Other	3

**Graph 1:**
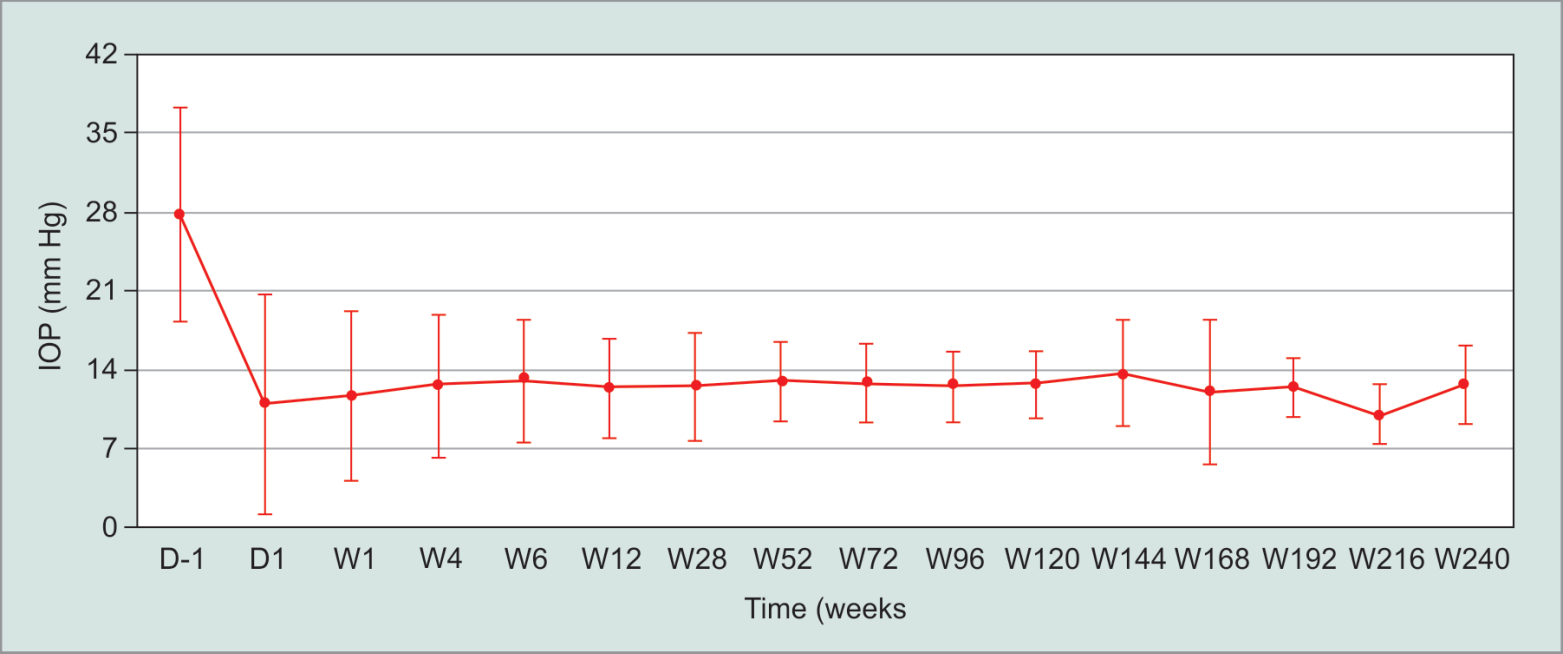
Intraocular pressure before and after DSMMC

The mean preoperatively IOP was 27.66 ± 9.47 mm Hg. There was a statistically significant IOP reduction post operation for up to 30 months and 48 months (*p* < 0.001). At 3, 12, 24 and 60 months post-operation, the IOP reduced by 54%, 54%, 55% and 54%, respectively. The mean IOP post-DS remained in the range 10 to 13 mm Hg (Graph 1).

**Graph 2:**
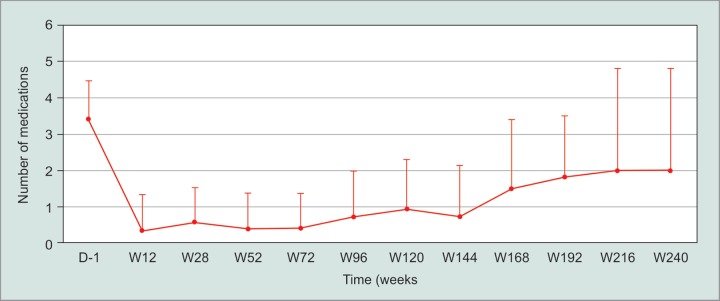
Medications before and after DSMMC

There was a statistically significant reduction in the number of medications required to control IOP post-DS to 30 months, *p* < 0.001 (Graph 2). The mean number of medications preoperatively was 3.35 ± 1.06 which reduced to 0.38 ± 0.97, 0.48 ± 0.98, 0.88 ± 1.27, 2 ± 2.83 at 3, 12, 24 and 60 months post operation.

**Graph 3:**
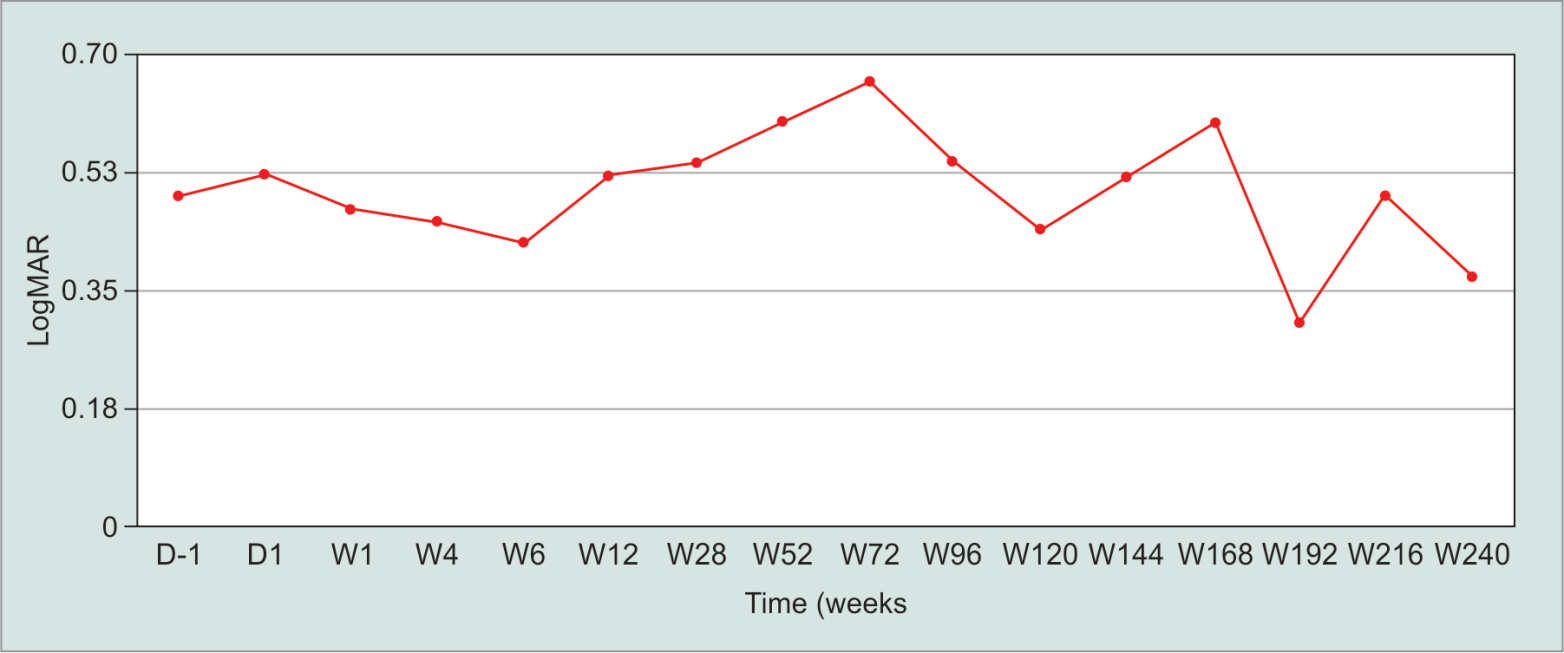
Mean best corrected visual acuity before and after DSMMC

The mean LogMAR BCVA preoperatively was 0.57 ± 0.42 and 0.53 ± 0.44 day one postoperatively. At week 12 it returned to preoperative level, the mean was 0.51, 0.60, 0.54 and 0.37 at 3, 12, 24 and 60 months respectively (Graph 3). There were no statistically significant differences between pre-operation and post-operation BCVA (*p* > 0.05).

**Graph 4:**
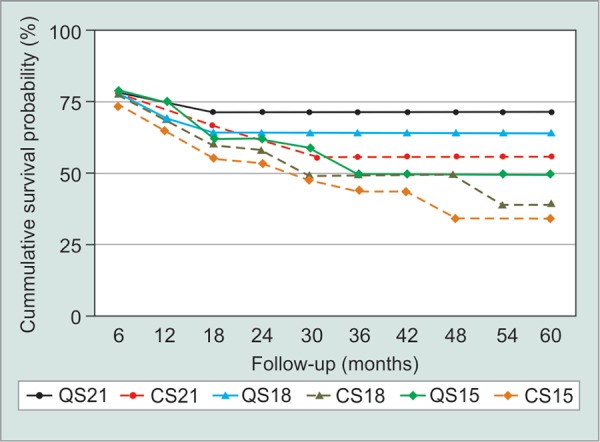
Kaplan-Meier cumulative probability curve for complete and qualified sucess for DSMMC

At 60 months, the Kaplan-Meier cumulative survival curve demonstrated that at < 21 mm Hg target IOP, CS and QS was achieved in 69.3% and 71.3%. At < 18 mm Hg target IOP, CS and QS was reached in 57.9% and 63.9% of eyes. Moreover, at < 15 mm Hg target IOP, CS and QS was attained in 45.2% and 48.7% of eyes (Graph 4).

Five surgeons (1 consultant and 4 fellows) performed the surgeries (79.8% by the consultant and 20.2% by fellows). There were no significant differences found in the cumulative survival rate for CS or QS for all three IOP criteria (*p* > 0.05). Similarly, no difference was found based on the patient's gender, age, type of glaucoma and intraoperative complications (*p* > 0.05). Table 2 summarises the intra- and post-operative complications. There were no patients with bleb-related endophthalmitis or surgery-induced cataract.

## DISCUSSION

This study has shown, for the first time, that DS is an effective and safe surgical treatment for OAG in Australian patients. In the short to medium term, IOP was significantly reduced with a low rate of complications during and after surgery.

Cilino et al.^10^ have also used MMC with Healon GV ophthalmic viscoelastic device (OVD), during DS, with comparable complete success for IOP < 21 mm Hg at 12 months to our study (78.9% *vs.* 71.8%), respectively. Beside using MMC and OVD to increase the success rates of DS, collagen implants (CI) have also been used. A randomized prospective study by Shaarawy et al. used contralateral eyes of 13 patients to compared between DSCI and without.^11^ Eyes with DSCI had better CS (69% *vs.* 38%) and QS (100% *vs.* 69%) compared to eyes without CI, respectively.^11^ It should be noted that the size of this study is small and the success rate in the non-implant group was low compared to other studies which may impact the comparison overall. Table 3 summarises studies with DSCI and goniopuncture used.^4,11–16^ The probability of complete success for IOP < 21 mm Hg was similar compared to our study. Furthermore, the percentage of eyes requiring goniopuncture post-DS were also similar, except for Mermoud et al. study where only 23% of eyes required goniopuncture post DS.^12^

**Table 2:** Intra- and post-operative complications (n = 99 eyes)

*Complications*	*Number*
Micro-perforation	11
Macro-perforation	1
Choroidal herniation	3
Retained viscoelastic	1
Hyphema*	5
Corneal epithelial defect*	2
*One patient had both hyphema and corneal epithelial defect	

Despite many studies, including this, demonstrating DS is effective at reducing IOP for OAG, TE remains the gold standard surgical procedure for glaucoma.^2^ This is perhaps because of its proven track record and well-documented efficacy as well as a perception that it is easier to learn than DS. There have been significant improvements in the safety of TE following the development of the Moorfields Safe Surgery System,^3^ but despite this, severe and potentially blinding complications can still occur. These include hypotony, flat or shallow anterior chamber, endophthalmitis, hyphaema, and choroidal detachment.^6,7^ An accurate understanding of the difference between the two techniques is best achieved by assessing studies that have compared them directly.

**Table 3:** Deep sclerectomy with collagen implant and goniopuncture: clinical results (listed by year of publication)

*Authors*	*Number of eyes*	*Mean follow-up months*	*Mitomycin (MMC)*	*% complete success (intraocular pressure <21 mm Hg)*	*Goniopuncture (%)*
Mermoud et al.	44	14.4 ± C6.3	NA	69	23
Karlen et al.	100	17.8 ± 8.7	NA	44.6	41
Ambresin et al.	20	24.3 ± 19.1	NA	40	45
Shaarawy et al.	52	44.5 ± 21	NA	64.3	48
Shaarawy et al.	105	64 ± 26.6	NA	57	51
Shaarawy et al.	13	49.5 ± 20	NA	69	46
Suominen et al.	15	12	0.4 mg/mL for 3 min	67	47
Our study	99	19.65 ± 14.11	0.2 mg/mL for 30–60 sec	55.6	49.5

A Cochrane review by Eldaly et al. compared the effectiveness of TE to non-penetrating trabecular surgery (DS and viscocanalostomy).^6^ There was no difference in the success of lowering IOP with or without drops for patients that underwent TE or DS (odd ratios (OR) 1.01, 95% Confidence interval (CI) 0.37 to 2.71). However, more reported complications were observed post-TE than post-DS (75.6 *vs.* 22.7%, respectively). Nearly 15.6% of TE participants experienced hypotony post-operation, while no cases of hypotony were reported in eyes that received DS. There were also more cases of a flat/ shallow anterior chamber (7.5 *vs.* 1.0%, respectively) and progressive cataract (11.9 *vs.* 2.0%, respectively) in TE participants compared to DS participants. These findings are consistent with our observed low postoperative complication rate with no cases of hypotony, flat/shallow anterior chamber or progressive cataract identified.

Although DS has lower complication rates compared to TE, it is less widely used by surgeons for many possible reasons including limited access to training, a perceived lack of efficacy and a perceived steep learning curve. The major intraoperative complication during DS is perforation of the trabeculo-descement's window during deep flap dissection with a reported rate of between 8 and 30% of cases in the early learning phase.^9,17^ While with further experience with non-penetrating surgery (NPS) the rate of trabeculodescemetic membrane rupture was reduced to 3–5%.^17^ Our study observed a low rate of perforation (10% micro-perforation and 1% macro-perforation) in the early cases. This number reduced to zero for later cases as proficiency with the surgical technique increased. Aslan et al. found similar rates of perforation for DS performed by an experienced consultant with 10.9% in early cases to 4.3% in late cases.^9^ They also found no significant differences between the perforation rate with different skilled operators (residents *vs.* consultant) (*p* = 0.28).^9^

Most papers in the literature describe a single surgeon, and many do not describe the expertise of the surgeon performing the procedure.^6,7,14,18–20^ Our study compared the outcome from surgeons with a variety of experience (consultant *vs.* fellows). Importantly there were no significant differences between the rate of success for all levels of IOP between the result performed by the consultant or the fellow. Aslan et al. evaluated the effects of DS performed by residents (group 1 has wet lab learning, and group 2 had direct guidance with the experienced surgeon) to experience surgeon. Each resident has completed a median of 50 TE.^9^ The CS at 6 months observed was 68.8% in group 1 residents, 62.5% in group 2 residents to 49.2% and 84.9% in the early and late cases of an experienced surgeon.^9^ DS is a safe procedure that can be implemented effectively and safely of surgeons with different experience especially if they are already competent in TE, but more importantly, it has fewer complications compared to TE.

A limitation of this study is the retrospective design. A small number of patients were lost to follow-up, and the method of MMC application was not equal for all patients. Another limitation of this study is the focus on clinical outcomes rather than patient-focused outcomes. As the quality of life measures was not routinely collected during patient follow-up, we were not able to determine from this study how surgery impacted patient quality of life. Regarding this particular issue, prospective studies with the quality of life analysis are required.

## CONCLUSION

In conclusion, DS is observed to be an effective surgical method with a favorable safety profile in obtaining IOP control in patients with OAG in the early to mid-term. It has a better safety profile compared to TE and can be performed by a surgeon of different experience safely and successfully.
